# Formation mechanism of binary complex based on β-lactoglobulin and propylene glycol alginate with different molecular weights: Structural characterization and delivery of curcumin

**DOI:** 10.3389/fnut.2022.965600

**Published:** 2022-07-19

**Authors:** Dongdong Lin, Jiaqi Su, Shuai Chen, Jiao Wei, Liang Zhang, Xiude Li, Fang Yuan

**Affiliations:** ^1^School of Physical Science and Technology, Qian Xuesen Collaborative Research Center of Astrochemistry and Space Life Sciences, Ningbo University, Ningbo, China; ^2^Beijing Advanced Innovation Center for Food Nutrition and Human Health, Key Laboratory of Functional Dairy, Ministry of Education, College of Food Science and Nutritional Engineering, China Agricultural University, Beijing, China; ^3^Particle and Interfacial Technology Group, Faculty of Bioscience Engineering, Ghent University, Ghent, Belgium; ^4^School of Public Health, Wuhan University, Wuhan, China; ^5^School of Food and Health, Beijing Technology and Business University, Beijing, China

**Keywords:** propylene glycol alginate, β-lactoglobulin, molecular weight, curcumin, formation mechanism, delivery system

## Abstract

The complexation of protein and polysaccharide has shown considerable potential for the encapsulation of functional food components. In this work, propylene glycol alginate (PGA) molecules with different molecular weights (100, 500, and 2,000 kDa) were prepared through H_2_O_2_ oxidation, which were further combined with β-lactoglobulin nanoparticles (β-lgNPs) to form PGA-β-lgNPs complexes for the delivery of curcumin (Cur). Results showed that the depolymerization of PGA molecule was resulted from the breakage of glycosidic bonds in the main chain, and the depolymerization rate of PGA molecule depended on the reaction time, temperature, solution pH and H_2_O_2_ concentration. As the increasing molecular weight of PGA, the particle size, zeta-potential and turbidity of the complexes were obviously increased. The formation of PGA/β-lgNPs complexes was mainly driven by non-covalent interaction, including electrostatic gravitational interaction, hydrogen bonding and hydrophobic effect. Interestingly, the difference in the molecular weight of PGA also led to significantly differences in the micro-morphology of the complexes, as PGA with a high molecular weight (2,000 kDa) generated the formation of a “fruit-tree” shaped structure, whereas PGA with relatively low molecular weight (100 and 500 kDa) led to spherical particles with a “core-shell” structure. In addition, the incorporation of PGA molecules into β-lgNPs dispersion also contributed to the improvement in the encapsulation efficiency of Cur as well as physicochemical stability of β-lgNPs, and PGA with a higher molecular weight was confirmed with a better effect. Findings in the current work may help to further understand the effect of molecular weight of polysaccharide on the physical and structural properties as well as effectiveness as delivery systems of polysaccharide-protein complexes, providing for the possibility for the design and development of more efficient carriers for bioactive compounds in food system.

## Introduction

Curcumin (Cur) is a hydrophobic bioactive polyphenolic compound isolated from the rhizome of turmeric, which has multiple biological and pharmacological activities such as antioxidant, antibacterial, anti-inflammatory, and anticancer (1, 2). Meanwhile, Cur has also shown potential therapeutic effects on a variety of chronic diseases, such as gastrointestinal and neurological diseases, diabetes and even cancer. Therefore, it has aroused strong research interests among researchers. Cur shows the highest stability under acid pH conditions ranged from 1 to 6. Unfortunately, it also has poor water solubility as well as extremely low *in vivo* bioavailability in this range. Although the water solubility of Cur increases when pH > 7, the stability decreases. Under the physiological pH condition (0.1 M phosphate buffer, 37^°^C, pH 7.2), 90% of Cur in a vitro formulation would be degraded within 30 min (3). In addition, Cur commonly shows high incompatibility with food matrix, making it difficult to be directly incorporated within food products. More importantly, it is also sensitive to extreme conditions such as light and heat, and therefore is easily degraded during processing and storage, resulting in the loss of color and biological activities. These properties severely limit the application of Cur in the food field as a food coloring or dietary supplement. An effective means of addressing this challenge is to encapsulate Cur within delivery systems, slowing down its chemical degradation, improving its water solubility and bioavailability, as well as providing the possibility to achieve controlled release (4–6).

In the past decade, food-grade colloidal nanoparticles have gained extensive attention as efficient delivery systems for the encapsulation of bioactive components due to their advantages of biodegradability, nontoxicity, and environmentally friendly (7). Among them, protein-based particles have been regarded as one of the most promising candidates. β-lactoglobulin (β-lg) is the main component of bovine whey protein, accounting for about 80% of whey protein. Owing to its remarkable affinity as well as biocompatibility, β-lg is regarded as an ideal natural carrier for a variety of functional hydrophobic components (8, 9). Numerous studies have shown that β-lg nanoparticles (β-lgNPs) can be formed through heating β-lg aqueous solution above its denaturation temperature (10, 11). The β-lgNPs usually have well-defined structures ranging from spherical to ellipsoidal with a hydrophobic cavity, endowing them with a higher encapsulation efficiency (EE; 12). However, the phenomenon of aggregation and sedimentation between β-lgNPs in dispersion is easy to occur, affecting the physical and chemical stability of the entire system during storage.

The incorporation of polysaccharide into β-lgNPs provides the possibility of the fabrication of a more stable delivery system. Protein/polysaccharide complexes arise primarily from electrostatic interactions between oppositely charged macromolecules. These interactions can induce the formation of various supramolecules such as complexes, aggregates and aggregates (13, 14). Through changing the conditions, complexes with different physicochemical and functional properties can be prepared, which significantly improve the encapsulation and stabilization efficiency of individual protein-based particles (15).

**FIGURE 1:**
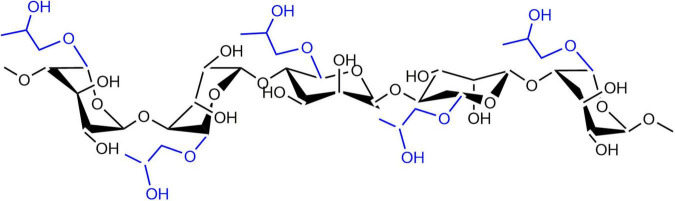
Chemical structure of PGA molecule.

Propylene glycol alginate (PGA) is a chemically modified anionic polysaccharide, which is produced through the esterification of alginic acid and propylene oxide. It is a high molecular weight linear polysaccharide which commonly linked by 31–65% β-D-mannuronic acid and 69–35% α-L-guluronic acid through 1,4 linked-glucosidic bands (Figure 1; 16). Chemically, PGA contains 50–85% of esterified carboxyl groups, making it an amphiphilic molecule with good surface-activity (17). The propylene glycol group in the PGA molecule is lipophilic, which can be combined with fat globules; while the uronic acid in the molecule is hydrophilic containing a large number of hydroxyl and carboxyl groups, which can interact with proteins (18). As a kind of food-grade polysaccharide, PGA can be used as an emulsifier and foaming agent due to its attractive properties including viscosity enhancement, stabilization, and film formation (19). In addition, the functional properties of proteins could be improved through combining proteins with PGA molecules (20, 21). For example, PGA can cause the destabilization and coagulation of casein micelles in milk, improve the thermal stability of β-lg, and affect the aggregation process of β-lg during thermal-induced gelation. Considering the fact that pure PGA is predominantly hydrophilic, combining PGA molecules with hydrophobic β-lg particles is expected to increase the hydrophilicity of the β-lg particles, thereby improving their solubility, as well as physicochemical stability.

Noteworthily, PGA with different molecular weights have different physicochemical properties and biological properties. The molecular weight of PGA depends on the number of basic units. On this account, for the purpose of acquiring low-molecular-weight PGA molecules, hydrogen peroxide (H_2_O_2_) degradation was used to reduce the polymerization degree (DP) of PGA in this study, since H_2_O_2_ could break down the oversized PGA molecules into appropriate size by acting on 1,4-glycosidic bonds, but hardly caused any infection in the ester groups on side-chain (22). To date, the development of protein- and polysaccharide-based delivery systems is one of the current research hotspots, as various kinds of delivery systems have been developed for the encapsulation of bioactive food ingredients. However, there are currently no report on the effects of molecular weights of polysaccharides on the properties and stability of on the β-lg-based complexes, which partly stimulates this work.

The present work was to explore the effect of PGA M (w) on the physicochemical properties, interaction mechanism and structural characterization of β-lg-PGA complexes as well as their EE to Cur. PGA with different M (w) were prepared through oxidative degradation by H_2_O_2_ under different conditions, during which the viscosity change was monitored to characterize the degradation behavior of PGA molecules. The depolymerization mechanism of PGA molecules was further explored through gel permeation chromatography (GPC), elemental composition analysis, and Fourier transform infrared spectroscopy (FTIR). β-lactoglobulin nanoparticles (β-lgNPs) fabricated through a thermally induced method were incorporated into PGA molecules to form PGA-β-lgNPs complexes. The interaction mechanism of PGA with different molecular weights and β-lgNPs and the formation rule of the complex were explored through the determination of the particle size, zeta-potential and turbidity. Fluorescence spectrum and scanning electron microscope (SEM) were also employed to characterize the structural properties of the complexes. The effects of the physicochemical properties of the complexes on the encapsulation and stability of Cur were systematically investigated. The PGA-β-lgNPs complexes were designed as a novel delivery vehicle of Cur, for the purpose of enhancing its anti-degradation stability. Results from this work would provide a new sight for the design and development of delivery vehicles for hydrophobic bioactive compounds.

## Materials and methods

### Materials

β-lactoglobulin was purchased from Davisco Foods International Inc. The reported composition (expressed as dry weight percent unless otherwise indicated) was: 97.6% protein, 0.4% fat, 2.0% ash, 4.0% moisture (all expressed as dry weight percent). High M (w; 2,000 kDa) of propylene glycol alginate (HPGA) with 88.2% esterified carboxyl groups was kindly donated by Bright Moon Marine SCI-Tech Co., Ltd. (Qingdao, China). Medium M (w; 1,000 kDa) of PGA (MPGA) and low M (w; 100 kDa) of PGA (LPGA) was prepared through a hydrogen peroxide hydrolysis method in our lab. Cur (98% purity) was purchased from China National Medicine Group (Shanghai, China). Dextran analytical standard were purchased from Sigma-Aldrich (St. Louis, MO, United States). Absolute ethanol (99.9%) was acquired from Eshowbokoo Biological Technology Co., Ltd. (Beijing, China). All other chemical reagents used in the present study were of analytical grade. Water purified by a Milli-Q system (Millipore, MA, United States) was used for all the experiments.

### Depolymerization reaction of propylene glycol alginate

The depolymerization reaction of PGA was carried out through an oxidation process. In brief, PGA powder was dissolved in deionized water (1.0 wt%), stirred continuously for at least 6 h and kept overnight at room temperature ensure complete dissolution. The PGA solution was pre-heated to 95^°^C, then a H_2_O_2_ solution was added to achieve a final H_2_O_2_ concentration of 0.05, 0.25, 0.5, 1.0, and 2.0 wt%, respectively, prior to heating in a water bath at 95^°^C for another 2 h. Samples were collected at designed time intervals of 20 min, cooling in an ice water in order to chill the reaction. Finally, solutions were adjusted to pH 4.0 with hydrochloric acid or sodium hydroxide. Samples were stored in the refrigerator at 4^°^C for further analysis in the form of liquid, part of them were frozen and dried with Alpha 1–2 D Plus freeze-drying apparatus (Marin Christ, Germany) for 48 h to obtain dry particles for solid state characterization analysis.

### Characterization of propylene glycol alginate with different molecular weight

#### Apparent viscosity

The apparent viscosity (η) of PGA samples was measured using a Brookfield DV-II+-Pro viscometer at 20^°^C. The PGA solutions were placed into a constant temperature detection chamber under a shear rate of 1.5 s^–1^. Data was recorded at a fixed interval of 20 min. The viscosity change was expressed as ln (η/η_0_), where the η_0_ is on behalf of the initial viscosity of PGA solution, whereas the η is the viscosity of PGA solution after H_2_O_2_ oxidation treatment.

#### Determination of molecular weight

The molecular weight of PGA after H_2_O_2_ hydrolysis was determined using a GPC equipped with a Waters 2414 differential detector (22). The chromatographic separation was performed on a TSK-GEL G-5000PWXL gel column and a G-3000 PWXL gel column in series. The PGA solutions (0.5 wt%) were injected and eluted by 0.1 M NaNO_3_ at a constant flow rate of 0.7 mL/min. Dextran standards of 1.27, 11.60, 5.22, 48.60, 147.60, 273.00, 409.80, and 667.80 kDa were used to obtain the calibration curve.

#### Analysis of constituent elements

The constituent elements of PGA samples were analyzed using a Vario EL cube elemental analyzer. 5.0 mg PGA powder was wrapped in a tin container and inserted in an autosampler vial tray, prior to placing into a combustion tube. Ultra-pure oxygen is introduced for combustion, and the generated N_2_, SO_2_, CO_2_, and H_2_O were detected by a thermal conductivity detector in turn. The final data is processed by a UNICUBE software to calculate the content of C, H, N, and S elements in samples.

#### Fourier transform infrared spectroscopy

Fourier transform infrared (FTIR) spectra of PGA molecules with different molecular weights were acquired using a Spectrum 100 Fourier transform spectrophotometer (Perkin-Elmer, United Kingdom) on the basis of a method described previously with sightly modifications (23). Briefly, 2.0 mg lyophilized PGA sample was mixed with 198 mg pure potassium bromide (KBr) powder, ground into fine powder in an agate mortar. Then the powder was compressed into a round crystal flake with a tablet machine and placed in a sample chamber for detection. Each sample was scanned for 64 times with a 4 cm^–1^ resolution for the wavenumbers ranging from 4,000 to 400 cm^–1^. The spectra obtained by scanning a crystal pellet prepared from pure KBr powder under the same conditions was used as a baseline. The acquired data were baseline corrected and analyzed using OMNIC 8.2 software (Thermo Nicolet, United States.).

### Preparation of β-lactoglobulin-propylene glycol alginate complexes with different propylene glycol alginate molecular weights

Propylene glycol alginate (0.25 *g*) with different molecular weights were dissolved in 100 ml deionized water. β-lg colloidal nanoparticles were prepared on the basis of the method presented by Schmitt et al. (11) with some modifications. β-lg solution (10 mg/mL) was firstly adjusted to pH 5.8 with 0.1N hydrochloric acid solution prior to heating in a water bath at 95^°^C for 5 min. After cooling down to the room temperature, the obtained β-lg colloidal particle dispersion was mixed with equal volume of PGA solution and the mixtures were re-adjusted to pH 4.0 to form the PGA-β-lg complexes. β-lg nanoparticles dispersion and PGA solution mixed with equal volume of deionized water were also obtained by the aforementioned process and used as the control sample. In this work, HPGA, MPGA, and LPGA were individually combined with β-lg nanoparticles and formed the complex, which were termed as HPGA-β-lg, MPGA-β-lg, and LPGA-β-lg, respectively.

### Physical and structural characterization of β-lactoglobulin-propylene glycol alginate complex

#### Particle size, size distribution, and zeta-potential measurements

Droplet size, size distribution and zeta-potential of the complex were determined by dynamic light scattering (DLS) using a Zetasizer Nano-ZS90 (Malvern Instruments, Worcestershire, United Kingdom) at a fixed detector angle of 90. Results were described as cumulants mean diameter (size, nm) for droplet size, polydispersity index (PdI) for particle size distribution and zeta-potential (mV) for particle electric charge date, respectively.

#### Nephelometry experiments

Turbidity of β-lg-PGA complex was determined using a HACH 2100N lab turbidimeter (Loveland, Colorado, United States). The calibration was performed with a Gelex^®^ Secondary Turbidity Standard Kit (HACH, Loveland, United States) formed by stable suspensions of a metal oxide in a gel. Measurements were carried out at ambient temperature (25^°^C).

#### Fluorescence measurements

Fluorometric experiments were carried out using a fluorescence spectrophotometer (F-7000, Hitachi, Japan) with an excitation wavelength of 280 nm. Scanning parameters were optimized with a slit width of 10 nm for both excitation and emission. The fluorescence emission spectrum monitored in the range of 290–450 nm with a scanning speed of 100 nm/min. Intrinsic fluorescence was measured at a constant β-lg concentration of 0.2 mg/mL. Each individual emission spectrum was the average of three runs and all data were collected at room temperature.

#### Scanning electron microscopy observation

The micromorphology of the samples was observed by a field emission SEM (SU8010, Hitachi) using an accelerating voltage of 5.0 kV (24). Lyophilized samples were adhered onto a specimen and sputter-coated with a gold layer to avoid charging under the electron beam.

#### Physical stability

The physical stability of samples was measured by LUMiSizer (L.U.M. GmbH, Berlin, Germany), an instrument utilizing centrifugal sedimentation to accelerate the occurrence of instability phenomena, such as sedimentation, flocculation or creaming (25). The integration graph was obtained through an enhanced optical system, which instantaneously measured the percentage of light transmittance as a function of time and position over the entire sample, the “creaming rate.” The instrumental parameters used for the measurement were as follow: temperature, 25^°^C; time Exp, 2,540 s; rotational speed, 4,000 rpm; number of times 255.

### Fabrication of curcumin-loaded ß-lactoglobulin-propylene glycol alginate complexes

Stock Cur alcoholic solution was prepared freshly at a concentration of 10 mg/mL by fully dissolving in absolute alcohol and then diluted by serial dilution to appropriate concentrations in alcohol before use. 1 mL of Cur solution with different concentrations was added to 10 mL β-lg-PGA complexes dispersion drop-by-drop under magnetic stirring. All samples were gently stirred for 0.5 h and then incubated at room temperature for at least 1 h before analysis.

### Physicochemical stability of curcumin-loaded ß-lactoglobulin-propylene glycol alginate complex

#### Entrapment efficiency and loading capacity

The EE and LE of Cur were determined according to the method of Gomez-Estaca et al. (26) with some modifications. Briefly, 1 mL of fresh sample was mixed with 4 mL of absolute ethanol through vortex oscillation for 2 min. Then the mixtures were centrifugated at a speed of 10,000 rpm for 30 min and the supernatants were collected. Absorbance at the wavelength of 426 nm was then measured using a UV-1800 spectrophotometer (Shimadzu Corporation, Kyoto, Japan). EE and LE were calculated by following the equations below:


$$EE\left(\%\right)=\frac{t\,o\,l\,a\,l\,C\,u\,r\,\text{-}\,f\,r\,e\,e\,C\,u\,r\,\left(m\,g\right)}{t\,o\,t\,a\,l\,C\,u\,r\,i\,n\,p\,u\,t\,\left(m\,g\right)}\times 100\%$$

$$LE\left(\%\right)=\frac{t\,o\,l\,a\,l\,C\,u\,r\,\text{-}\,f\,r\,e\,e\,C\,u\,r\,\left(m\,g\right)}{t\,o\,t\,a\,l\,a\,m\,o\,u\,n\,t\,o\,f\,P\,G\,A\,a\,n\,d\,\beta \,\text{-}\,l\,g\,i\,n\,p\,u\,t\,\left(m\,g\right)}\times 100\%$$

#### Photochemical stability

The photochemical stability of complexes was evaluated according to the method described in a previous report using a controlled light cabinet (Q-SUN Xe-1 Xenon Test Chamber, Q-Lab, United States; 27). Samples were placed in transparent glass bottles, which were flushed with nitrogen to avoid the existence of oxygen. The light intensity was set to 0.68 W/m^2^ and the temperature was controlled with a maximum value of 45^°^C. Solutions were sampled at a fixed interval of 20 min. The Cur content was quantified with the method described in 2.7.1 and expressed as relative content (RC, %), which was calculated according to the formula below:


$$RC\left(\%\right)=\frac{C}{C_{0}}\times 100\%$$

where *C*_0_ and *C* were the initial and retained concentrations of the Cur, respectively.

#### Thermal stability

Samples were stored under 85^°^C for 180 min. The Cur content were estimated at fixed time intervals with the methods described in section “Entrapment efficiency and loading capacity.”

### Statistical analysis

Samples were prepared in duplicate, and measurements were performed in triplicate. Data were analyzed using the software package SPSS 18.0 (SPSS Inc., Chicago, United States). Results were reported as the mean value and standard deviation of two separate injections. Statistical differences were determined by one-way analysis of variance with Duncan procedure, and differences of main effects were identified to be significant with *p* < 0.05.

## Results and discussion

### Factors affecting the depolymerization of propylene glycol alginate

**FIGURE 2:**
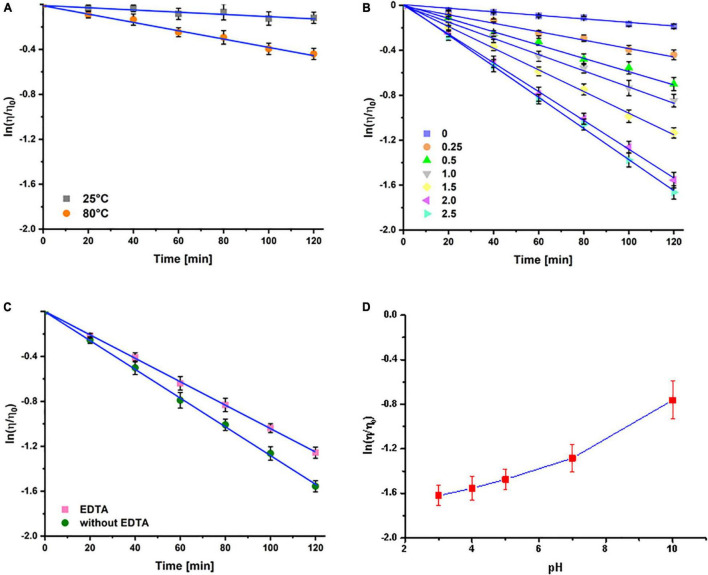
Effect of temperature **(A)**, H_2_O_2_ concentration **(B)**, EDTA **(C)**, and pH **(D)** on the depolymerization degree of PGA molecules. This figure adopted from 41.

The degree of depolymerization of 2.0 wt% PGA molecules under different reaction conditions was assessed through viscosity measurements. As shown in Figure 2A, the ln(η/η_0_) value of PGA solution significantly decreased as the increasing reaction time, exhibiting typical kinetic reaction characteristics of primary degradation. It can be also observed that the ln(η/η_0_) value of PGA solution decreased to –0.12 after the addition of 0.25 wt% H_2_O_2_ solution for at room temperature (25^°^C), and the viscosity of PGA solution decreased by about 11.3%; whereas the ln(η/η_0_) value of PGA solution decreased rapidly to –0.44 when placing in a water bath heating at 80^°^C, and the viscosity of PGA solution decreased about 35.7%. This indicated that the PGA depolymerization process had intensively influence on the reaction temperature, and the higher the temperature, the greater the degradation rate. Figure 2B shows the variation curves of ln(η/η_0_) value for PGA solutions treated with different concentrations of H_2_O_2_ as a function of time. Apparently, the ln(η/η_0_) value of all the samples treated by gradient concentrations of H_2_O_2_ solutions under different temperatures showed a tendency of decrease as the increasing treatment time, revealing that the PGA molecules were subjected to different degrees of degradation. Without the addition of H_2_O_2_ solution, the apparent viscosity of PGA solution was slightly changed after 2 h of treatment, suggesting that the effect of individual heat-treatment had little influence on the depolymerization rate of PGA; on the contrary, the depolymerization rate of PGA was mainly influenced by the concentration of H_2_O_2_ solution in the system. As desired, when the H_2_O_2_ concentration was between 0.25 and 2.5 wt%, the H_2_O_2_ concentration was positively correlated with the ln(η/η_0_) value of PGA under the same temperature conditions, which signified that the increase in the H_2_O_2_ concentration in the system facilitated the depolymerization of PGA molecules. It could be also observed that and the depolymerization rate was obviously increased with the elevating H_2_O_2_ concentration. However, with the addition of 2.0 wt% H_2_O_2_, the ln(η/η_0_) ratio of PGA solution decreased to –1.57 after 2 h of reaction, whereas the ln(η/η_0_) of PGA solution was –1.66 under the treatment of 2.5 wt% H_2_O_2_ for 2 h. These results indicated that when the H_2_O_2_ concentration was higher than 2.0 wt%, the increase in the H_2_O_2_ concentration had less impact on the depolymerization rate of PGA molecules. Therefore, 2.0 wt% H_2_O_2_ solution was chosen for subsequent experiments.

Interestingly enough, the addition of EDTA into PGA solutions resulted in an obvious decrease in the reduction rate of ln(η/η_0_) value (Figure 2C). We therefore hypothesis that the presence of metal ions in the system might exert a catalytic effect on the depolymerization of PGA. The depolymerization of PGA induced by H_2_O_2_ is mainly initiated by free radicals. Since traces of metal ions were existed in PGA samples, free radicals with high activity were therefore generated through the depolymerization of PGA catalyzed by these metal ions. The resulting free radicals are strong oxidizing agents that have the ability to destroy the glycosidic bonds and extract hydrogen atoms from the glycosidic bonds of PGA, leading to the molecular rearrangement of PGA. On the other hand, the above hypothesis was further verified by the changing tendency in the depolymerization rate of PGA. As shown in Figure 2D, the depolymerization rate was significantly decreased with the increasing pH. The most likely explanation is that the effective binding of metal ions to H_2_O_2_ molecules can only be achieved under acidic conditions; while the ionization reaction of H_2_O_2_ is intensified under alkaline conditions, thus reducing its oxidation activity.

### Physical and structural properties of propylene glycol alginate with different molecular weights

The changes in the composition and structure of PGA after H_2_O_2_ treatment were analyzed by a combination of various characterization methods. The main properties of natural PGA and oxidized PGA are summarized in Table 1. The molecular weight of PGA under different oxidative conditions was determined by GPC. Results showed that the molecular weight of PGA samples treated with 2.0 wt% H_2_O_2_ solution at 80^°^C for 40 min (MPGA) and 120 min (LPGA) decreased from the initial 2,000 kDa to 1,000 kDa and 100 kDa, respectively. In addition, the elemental composition of the oxidized PGA had not significant changes, but the mass ratio of C/H was slightly increased. The freeze-dried oxidized PGA sample was tested with starch-potassium iodide test paper, and it was found that there was no H_2_O_2_ residue inside the oxidized samples. This finding might be explained by the possibility that part of H_2_O_2_ was consumed by modifying the chemical structure of PGA, while another part of H_2_O_2_ may be thermally resolved during the oxidation process. Besides, the pH value of the PGA solution was also dramatically decreased, which was mainly due to the interruption of the main chain and the formation of carboxylic acid during the oxidation process of PGA.

**TABLE 1:** Main properties of PGA before and after H_2_O_2_ oxidation.

Sample name	Molecular weight (kDa)	pH	Element composition				
			C%	H%	C/H	N	S
HPGA	2,000	4.00	33.52	6.361	5.27	0.45	0.392
MPGA	1,000	3.78	33.40	6.193	5.39	0.39	0.248
LPGA	100	3.52	33.23	6.137	5.41	0.27	0.236

**FIGURE 3:**
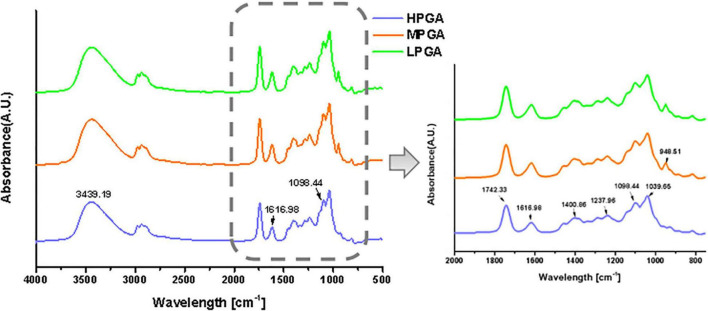
FTIR spectra of PGA molecules with different M (w).

Fourier transform infrared spectroscopy was used to determine the intramolecular interactions within PGA with different molecular weights. Figure 3 show the FTIR of PGA before and after H_2_O_2_ treatment. In the spectra of HPGA, the characteristic peaks of were located at 3,439 cm^–1^ (C-H stretching of unsaturated carbon), 1,742.33 cm^–1^ (-C = O stretching of ester group), 1,237.96 cm^–1^ (C-C-H and O-C-H stretching), 1,098.44 cm^–1^ (C-O and C-C stretching of the pyranose ring), and 1,039.65 cm^–1^ (C–O stretching), respectively. In addition, the asymmetric and symmetric stretching of the carboxylate vibrations appeared at 1,616.98 cm^–1^ and 1,400.86 cm^–1^, respectively. The FTIR spectra of MPGA and LPGA still maintained most of the characteristic adsorption peaks of HPGA, however, there were also some differences. For example, the peaks at 1,616.98 cm^–1^ and 1,098.44 cm^–1^ were broadened and slightly blue-shifted. In addition, a new absorption peak appeared at 948.51 cm^–1^. The former indicated the formation of carboxyl groups in PGA molecules, while the latter could be attributed to the C–O stretching vibration of uronic acid residues (22). These phenomena were consistent with the speculation in a previous report that H_2_O_2_ destroyed only the glycosidic bond during oxidation, leading to the breakage of the PGA backbone and the formation of -COOH groups, thus changing the structure of the terminal residue.

### Particle size, polydispersity index, zeta potential, and turbidity of complexes

**FIGURE 4:**
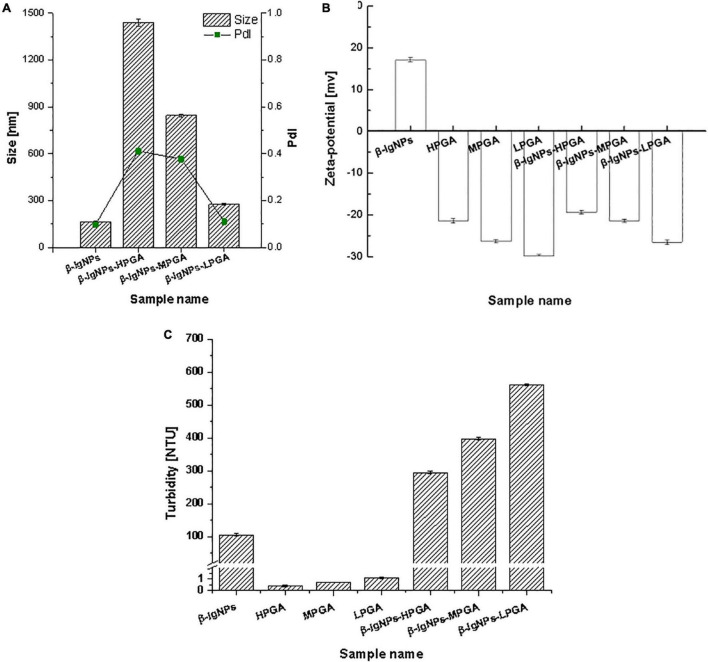
Particle size, PDI **(A)**, zeta-potential **(B)** and turbidity **(C)** of β-lgNPs and PGA-β-lgNPs complexes with different M (w).

The particle sizes of the complexes formed by β-lgNPs and PGA with different molecular weights are shown in Figure 4A. The average particle size of the individual β-lgNPs was 164.2 nm with a PdI value of 0.167, indicating that the size distribution of β-lgNPs was highly concentrated. After incorporating with PGA of different molecular weights, the particle size of complex samples was significantly increased, which might be explained by the fact that positively charged β-lgNPs was able to combine with anionic PGA to form PGA/β-lgNPs complexes under acidic conditions. With the increasing molecular weight of PGA, the particle size of resulting LPGA-β-lgNPs, MPGA-β-lgNPs and HPGA-β-lgNPs was increased to 276.4, 844.6, and 1437.8 nm, respectively. This was mostly due to the larger spatial volume of PGA molecules led by the increase in the molecular weight of PGA, which further resulted in the formation of complexes with a larger particle size. The zeta-potential of complexes formed by β-lgNPs and PGA with different molecular weights was shown in Figure 4B. The zeta-potential of β-lgNPs was +17.4 mV at pH 4.0, which might be ascribed to the positive charge of β-lg when the pH is below its isoelectric point (pI = 4.7). When PGA with different molecular weights were introduced, the zeta-potential of the resulting complexes were dramatically decreased below –20 mV, which was closed to those of corresponding individual PGA molecules, indicating that the overall charge of complexes was primarily dominated by anionic PGA molecules through electrostatic interactions. The electrostatic attraction between amino acid residue –NH^3+^ in β-lg molecules and -COO in PGA molecules was considered to be the main driving force for the formation of PGA/β-lgNPs complexes. This phenomenon is similar to complexes formed by zein and PGA (28), as well as lactoferrin and carrageenan (29).

As shown in Figure 4C, the turbidity values of PGA/β-lgNPs complexes (295.0∼562.0 NTU) were much higher than those of single β-lgNPs (105.9 NTU) and PGA solutions (0.4∼1.1 NTU). It was also noteworthy that the turbidity of PGA/β-lgNPs complexes showed a similar changing tendency with particle size. Theoretically speaking, during the process of the turbidity measurement, after a parallel light beam from a light source passed through complex samples, part of the light is absorbed and scattered by the particles, while the other part passes through the dispersion. The intensity of the scattered light is proportional to the turbidity of the testing samples, then the turbidimeter calculates automatically the turbidity value from the intensity of the scattered light. Therefore, the change of particle size and turbidity of particle dispersion normally shows a certain extent of correlation. This result further confirmed that β-lgNPs were able to combine with the PGA with different molecular weights to form complexes with different sizes. To sum up, the turbidity of PGA/β-lgNPs complexes was affected by the particle size, which was dominated mainly through the electrostatic interactions between PGA molecules and β-lgNPs.

### Fluorescence spectroscopy

Fluorescence spectroscopy is an effective way to investigate the structural transition and binding properties of protein with biological macromolecules, like polysaccharides, and small molecules such as Cur (30), retinol and retinoic acid (8) and riboflavin (31). The tryptophan (Trp) residues are highly sensitive to the local environmental conditions of proteins, and the conformational changes of proteins can be therefore monitored by observing changes in tryptophan fluorescence. The fluorescent tryptophan residues in β-lg, Trp-19 and Trp-61, are responsible for the intrinsic fluorescence of β-lg. Trp-19 is in a non-polar environment and contributes to 80% of total fluorescence, while Trp-61 is partly exposed to aqueous solvent and has minor contribution to Trp fluorescence (32). When other molecules interact with β-lg, tryptophan fluorescence may change according to the impact of such interaction on the protein conformation (33, 34).

**FIGURE 5:**
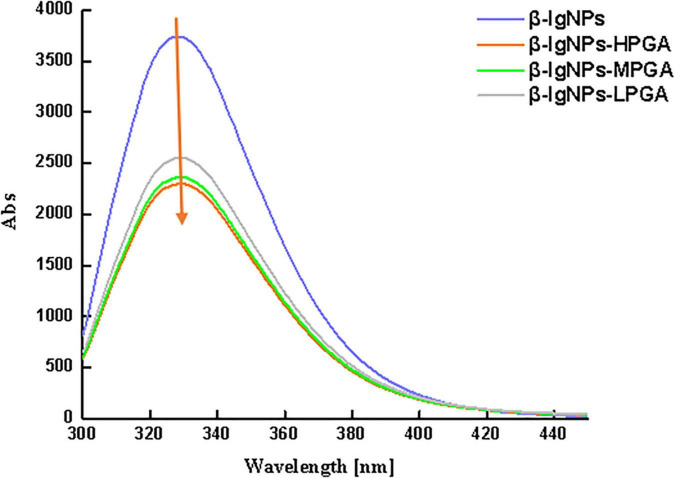
The fluorescence spectroscopy of β-lgNPs and PGA-β-lgNPs complexes with different PGA M (w).

Considering the fact that the interaction between β-lgNPs and PGA molecules is highly possible to cause the change of Trp fluorescence, the fluorescence spectroscopy were employed in order to further analyze the interactions. Figure 5 shows the effect of the addition of PGA molecules with different molecular weights on the fluorescence intensity of β-lgNPs. It could be found that β-lgNPs exhibited a strong fluorescence emission peak at 304 nm when it was excited at 280 nm, which was consistent with the previous report. The fluorescence intensity of individual PGA molecules is close to 0 and can be ignored. When β-lgNPs were combined with PGA molecules, although no shift in the emission peak could be observed, the height of the emission peak was obviously decreased as the increasing PGA molecular weight. In general, Trp residues are usually partially or fully buried in the hydrophobic core of proteins due to their hydrophobicity. The unfolding of protein would result in the exposure of hydrophobic amino acid residues that were originally located on the inside, whereas the aggregation of protein molecules generally leads to the shielding of hydrophilic amino acid residues that were originally located outside (29). Proteins unfold and rapidly aggregate to form new aggregates during the particle formation process, resulting in the exposure of more hydrophobic groups. In this case, the combination of positively charged β-lgNPs combined to anionic PGA through electrostatic attraction interaction resulted in the shielding of tryptophan residues and the decrease in fluorescence intensity. Perez et al. found that the addition of λ-carrageenan resulted in a decrease in the fluorescence intensity of whey protein concentrates (35). At the same time, the decrease in the intensity of β-lg fluorescence spectra was also observed in β-lgNPs/chitosan complexes, which might be due to the combination of chitosan and β-lgNPs through electrostatic interaction to form complexes (36). In addition, with the increase in the molecular weight of PGA, the fluorescence intensity of β-lgNPs was significantly decreased. This might be due to the interaction between LPGA and the hydrophilic groups on the surface of β-lgNPs, which facilitated the movement of Trp residues in β-lgNPs toward the hydrophobic interior. Thus, nanoparticles with a shell-core structure were formed. On the contrary, when HPGA bound to β-lgNPs, the fluorescence intensity was further decreased, which might be related to the aggregation and adhesion of β-lgNPs onto the long chains of PGA molecules. Chen et al. (37) reported the shielding phenomenon of Trp residues caused by protein aggregation, where hyaluronic acid and sodium hyaluronate with a high molecular weight and can lead to the aggregation of zein particles, thereby reducing their fluorescence intensity.

### Microstructure

**FIGURE 6:**
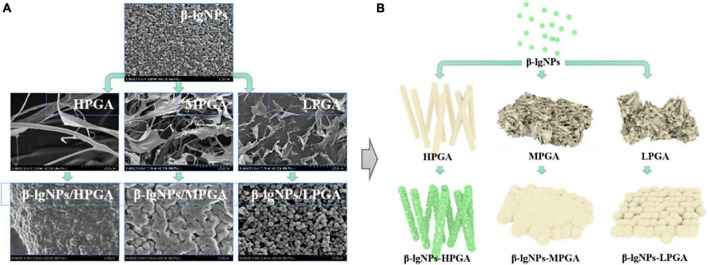
SEM images **(A)** and schematic representation of the formation mechanism **(B)** of β-lgNPs, PGA and PGA-β-lgNPs complexes with different PGA M (w).

The micro-morphology of β-lgNPs, PGA with different molecular weights and PGA-β-lgNPs complexes were observed by a cryo-scanning electron microscopy (Figure 6A). Individual β-lgNPs usually exhibited a regular spherical shape with smooth surface and uniform particle size distribution, which was consistent with the results of DLS. However, PGAs with different molecular weights showed obvious differences in their morphologies. LPGA, MPGA, and HPGA presented as sawdust flakes, short trunks and long filament meshes, respectively. In addition, the complexes formed by the combination of β-lgNPs and PGA with different molecular weights also showed different microstructures. The LPGA-β-lgNPs showed a clear spherical shape, accompanied by a larger particle size and a rougher surface compared to those of individual β-lgNPs. The particle size significantly increased when the molecular weight of PGA increased from 100 to 1,000 kDa, as evidenced by the DLS results. Besides, it was hard to identify an individual particle due to the ambiguous boundaries between adjacent particles, which might be ascribed to the introduction of MPGA molecules, which led to the adhesion of β-lgNPs onto MPGA molecules, and further, the formation of larger aggregates. Interestingly, when the molecular weight of PGA increased to 2,000 kDa, it could be found that spherical β-lgNPs could still be adsorbed to the surface of HPGA through electrostatic attraction.

On the basis of the above-mentioned analysis, a speculative schematic was presented in order to illustrate the possible structures and formation mechanisms of complexes formed by β-lgNPs and PGA with different molecular weights. As depicted in Figure 6B, for LPGA-β-lgNPs and MPGA-β-lgNPs, core-shell structured composite particles were fabricated with the adsorption of negative-charged PGA onto the surface of positive-charged β-lgNPs. The distinction was that the excessive length of PGA molecules facilitated the interpenetration and entanglement among PGA molecules, which resulted in a blurring boundary between particles. Unlike them, HPGA-β-lgNPs exhibited a more special structure with β-lgNPs hung on the “trunk” of HPGA like “fruits.”

### Physical stability

**FIGURE 7:**
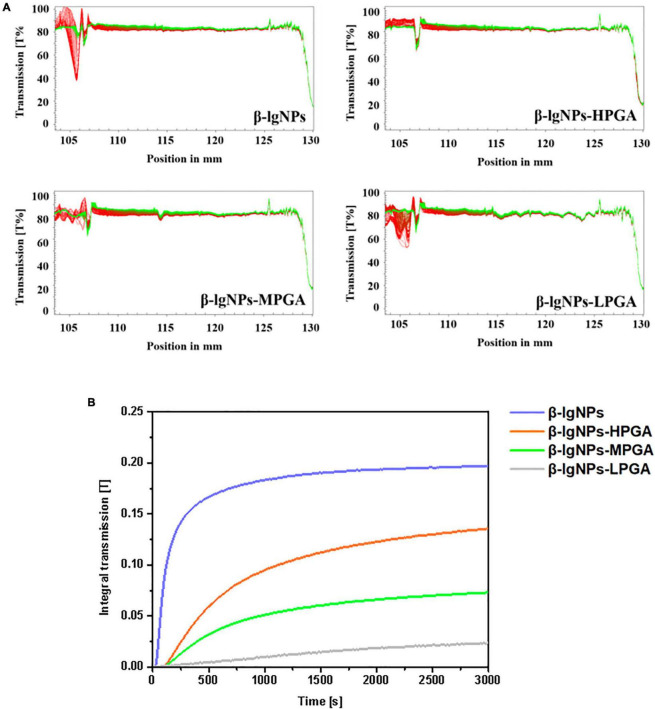
The integral transmission **(A)** and time-dependent integral transmission profiles **(B)** of PGA-β-lgNPs complexes with different PGA M (w) as a function of time.

The physical stability of complex dispersions formed by β-lgNPs and PGA with different molecular weights were assessed using LUMiSizer. In general, the smaller the normalized transmittance (%), the lower the aggregation degree of the complex samples and the more stable the sample under centrifugal force (38). Figure 7A showed the time-dependent integral transmission profiles at different positions (h) of complex samples under centrifugal conditions for a visual comparison. In these profiles, the position at 105 mm is on behalf of the bottom of the sample, whereas the position at approximately 130 mm corresponds to the top of the sample. It could be found that the light transmission of individual β-lgNPs showed a remarkable decrease as the increasing detection time. On the contrary, the changes in the light transmission of all PGA-β-lgNPs complex samples were not significant throughout the centrifugal process, indicating that these complex dispersions had a relatively high physical stability. This was mostly due to the negative charge of PGA chains, which were able to lead to the electrostatic repulsion between complexes, thereby avoiding the occurrence of instability phenomena such as aggregation or sedimentation. For the purpose of further comparing the difference in the centrifugal stability of complex samples, the integral transmittance (%) curve versus time was also calculated using the LUMiSizer analysis software, and results were shown in Figure 7B. The lower the slope of the curve, the higher the stability of the sample. The slope of the curve was increased as the increasing molecular weight of PGA. It was worth noting that the slope of the light transmission curve of LPGA-β-lgNPs complex dispersion was lower than that of other samples, indicating that the LPGA-β-lgNPs complex had the highest centrifugal stability. Two speculations might account for this phenomenon. Firstly, the existence of LPGA led to a higher viscosity of the complex dispersion, which hindered the movement and close approach of β-lgNPs in the dispersion. In addition, the LPGA-β-lgNPs was determined with the highest surface charge, therefore the electrostatic repulsion prevented the aggregation of protein particles, promoting the increase in the centrifugal stability of the complex dispersion.

### Encapsulation efficiency and loading capacity

The effects of different PGA molecular weights on the encapsulation efficiency (EE) and loading capacity (LC) of PGA-β-lgNPs complexes for the delivery of Cur were shown in Table 2. The EE of β-lgNPs-Cur was 44.60%, which was much lower than that of β-lgNPs-PGA complexes. The EE of the PGA-β-lgNPs complexes for Cur was gradually elevated with the increasing PGA molecular weight. The encapsulation rate of β-lgNPs-HPGA was 94.02%, and the loading amount was up to 12.52%. These results showed that introducing PGA molecules into β-lgNPs to form PGA-β-lgNPs complex could greatly improve the EE and LC of β-lgNPs for the encapsulation of hydrophobic Cur. This was consistent with the previous report by Sun et al. (39), who had found that the EE of Quercetagetin was significantly improved after zein was combined with PGA. In conclusion, the β-lgNPs-HPGA complexes showed remarkable properties and good application prospects as delivery vehicles.

**TABLE 2:** Entrapment efficiency (EE) and loading capacity (LC) of Cur in PGA-β-lgNPs complexes.

Sample name	EE%	LC%
β-lgNPs	44.60	8.92
β-lgNPs-HPGA	94.02	12.52
β-lgNPs-MPGA	89.69	11.93
β-lgNPs-LPGA	86.11	11.45

### Photochemical and storage stability

**FIGURE 8:**
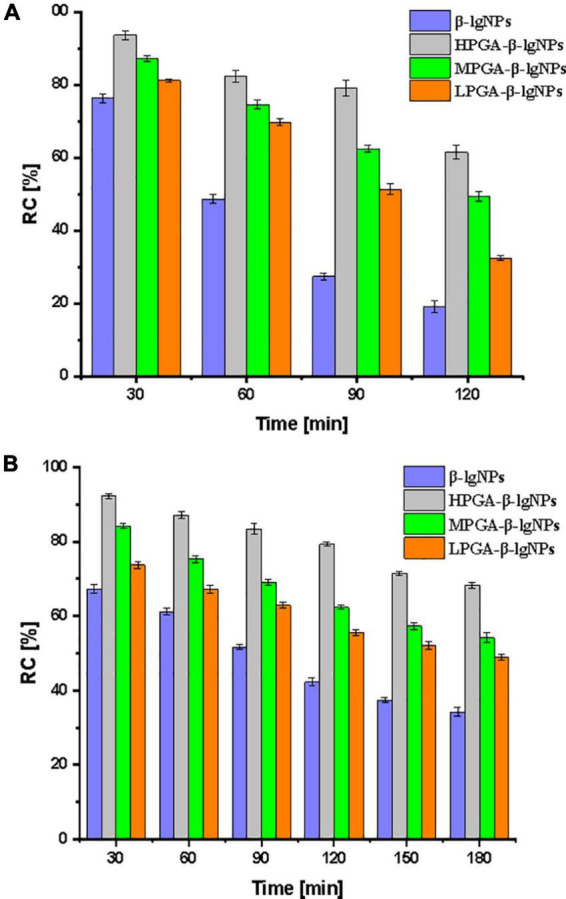
Relatively content (RC%) of curcumin in PGA-β-lgNPs complexes during the exposure of UV light for 120 min **(A)** and heat treatment for 180 min **(B)**.

The effective content of nutraceuticals in food products is dramatically affected by the external environmental conditions (40). Dur to the light- and heat- sensitive characteristics of Cur, Cur-loaded complex samples were subjected a UV-light irradiation and an accelerated storage test under 85^°^C for 180 min, in order to assess the effectiveness of different formulations as carriers for the delivery of Cur. The photo degradation of Cur in samples was shown in Figure 8. The RC% value of Cur encapsulated within the PGA-β-lgNPs was significantly higher than that of Cur in individual β-lgNPs (46.2%), suggesting a better protective effect of PGA-β-lgNPs complexes compared to the individual β-lgNPs on Cur. It could be also found that MPGA-β-lgNPs exhibited the highest retention rate of Cur compared to the other complexes.

On the other hand, a thermal accelerated test was also performed on the complexes samples in order to assess their long-time storage stability as well as predict their shelf life, since the shelf life play a very important role on the commercialization of functional foods. After a period of 2-weeks storage under 55^°^C, The RC% value was 39.6, 44.8, 59.2, and 67.4%, for β-lgNPs, LPGA-β-lgNPs, MPGA-β-lgNPs, and HPGA-β-lgNPs, respectively. Again, MPGA-β-lgNPs showed the best protective effect on Cur when exposed to UV light. The extremely high retention rate of Cur within MPGA-β-lgNPs could be attributed to the microstructure of MPGA-β-lgNPs. Unlike HPGA-β-lgNPs, where β-lgNPs were adsorbed onto the main-chain of PGA molecules, which might result in the exposure of Cur-loaded β-lgNPs to external environments. On the contrary, β-lgNPs were entirely embedded within the network framework formed by PGA molecules in LPGA-β-lgNPs and MPGA-β-lgNPs, especially for MPGA-β-lgNPs, in which the overlong PGA molecules located at the outer layer of β-lgNPs interpenetrated and entangled with each other (24, 39). These results highlight that the combination of PGA to β-lgNPs was responsible for the improved protection effect of β-lgNPs on Cur against unfavorable conditions.

## Conclusion

In conclusion, PGA with different molecular weights were fabricated through H_2_O_2_ oxidation to form PGA-β-lgNPs as carriers for the delivery of Cur. The depolymerization of PGA molecule was triggered by the breakage of glycosidic bonds in the main chain, which was mainly influenced by the reaction time, temperature, solution pH and H_2_O_2_ concentration during the oxidation process. The combination of PGA with different molecular weights (100, 500, and 2,000 kDa) with β-lgNPs were confirmed mainly through electrostatic gravitational interaction, hydrogen bonding and hydrophobic effect. With the increase in the molecular weight of PGA, the particle size, zeta-potential and turbidity of complexes were significantly increased, whereas the fluorescence intensity was gradually decreased. The PGA-β-lgNPs complexes exhibited different micro-morphologies mainly depending on the molecular weight of PGA. Complexes formed between β-lgNPs and LPGA and MPGA were existed as spherical particles, whereas HPGA/β-lgNPs complex were in a “fruit-tree” shape with PGA molecules acted as the tree trunk and β-lgNPs served as the fruits. The formation of PGA-β-lgNPs complexes could not only contributed to increase the EE of Cur, but also effectively retarded the degradation of Cur when exposed to either UV light or high-temperature storage. In addition, the increase in the molecular also significantly promoted the improvement in the physicochemical stability of the complexes. These findings indicated that PGA-β-lgNPs complexes, especially HPGA-β-lgNPs complex, would be a promising carrier for the encapsulation of hydrophobic functional components.

## Data availability statement

The original contributions presented in this study are included in the article/supplementary material, further inquiries can be directed to the corresponding authors.

## Author contributions

DL: methodology and writing—original draft preparation. JS: conceptualization, visualization, and investigation. SC: visualization and investigation. JW: software. LZ: data curation. XL: visualization. FY: writing—reviewing and editing. All authors contributed to the article and approved the submitted version.

## Conflict of interest

The authors declare that the research was conducted in the absence of any commercial or financial relationships that could be construed as a potential conflict of interest.

## Publisher’s note

All claims expressed in this article are solely those of the authors and do not necessarily represent those of their affiliated organizations, or those of the publisher, the editors and the reviewers. Any product that may be evaluated in this article, or claim that may be made by its manufacturer, is not guaranteed or endorsed by the publisher.
